# Photoelectron Spectroscopy of OH^−^-Anion–Water Clusters Generated by Ultrasonic Nebulizer

**DOI:** 10.3390/ijms23084175

**Published:** 2022-04-10

**Authors:** Minchae Kang, Chang Jun Park, Hyung Min Kim, Sang Hak Lee

**Affiliations:** 1 Department of Chemistry, Pusan National University, Busan 46241, Korea; wndud0621@naver.com (M.K.); mc_ccoli1995@naver.com (C.J.P.); 2 Department of Applied Chemistry, Kookmin University, Seoul 02707, Korea; hyungkim@kookmin.ac.kr

**Keywords:** anion clusters, ultrasonic vibrator, gas phase, hydroxide photoelectron spectroscopy, mass spectroscopy

## Abstract

Investigating molecules in the gas phase is the only way to discover their intrinsic molecular properties; however, it is challenging to produce the gaseous phase of large-molecule chemicals. Thermal evaporation is typically used to convert molecules into gases, but it is still challenging to study ionic molecules in solutions in the gas phase. Electrospray ionization is one of the best methods to generate molecules in the gas phase, and it is uniquely capable of studying large biomolecules, including proteins. However, the molecular temperature required to study the spectroscopic properties of the molecules is very high. In this study, we developed a new, simple evaporation method using an ultrasonic nebulizer to obtain gas-phase molecules. Using this new equipment, we observed OH^−^ anions and their water clusters in the gas phase and obtained their photoelectron spectra. We observed that the vertical electron-detachment energy (VDE) of OH^−^ was 1.90 ± 0.05 eV and the VDEs of its water clusters and OH^−^ (H_2_O)*_n_* (*n* = 1–2) decreased to 1.50 ± 0.05 eV (*n* = 1) and 1.30 ± 0.05 eV (*n* = 2), respectively.

## 1. Introduction

Gas-phase spectroscopy is a unique method for investigating the intrinsic properties of molecules [1,2,3,4,5]. Researchers have extensively studied the optical, structural, and electronic properties of small molecules in the gas phase. Small molecules are the building blocks of larger molecules and materials [6,7,8]. Therefore, it is critical to gain fundamental knowledge about small molecules in order to increase our understanding of biological systems and to achieve control of material properties [9,10]. For example, protein-folding and phase-separation phenomena in cells vary depending on their basic molecular properties and interactions [10,11]. 

The study of molecular properties using spectroscopy in the gas phase has the advantage of eliminating the effect of the perturbation of other molecules. However, producing gas-phase molecules is challenging. In general, small molecules are converted to the gas phase by heating the samples, which is relatively easy for molecules with low boiling points (<200 °C). High-pressure inert gases, such as Ne or Ar, can then be applied to create supersonic expansion, which in turn reduces the molecular temperature and helps in the formation of the molecular ground state and neutral, cationic, and anionic ground states [1,2,3,4].

When large molecules such as peptides, DNA, RNA, and proteins are heated for vaporization, they can easily fragment because of their high internal energy. This makes it difficult to study the parent molecules in the gas phase via heating. Alternatively, mild evaporation and ionization methods are required in order to produce gas phases of large biomolecules. Many researchers have attempted and reported soft evaporation and ionization methods, including electrospray [12], laser desorption [13] and MALDI [14], which to some extent have facilitated the study of larger molecules in the gas phase. Among these, laser desorption and MALDI are not suitable for evaporating ionic chemicals in solution because they require solid-state samples before changing to the gas phase. Instead, electrospray ionization is used to directly convert molecules in solution into the gas phase; therefore, it is the most common method for studying large biomolecules such as RNA, DNA, and proteins in the gas phase [15,16]. However, electrospray ionization is difficult to design and highly sensitive to RF frequency; therefore, researchers have frequently attempted to optimize the conditions of the equipment in various ways, depending on the samples. In addition, because it does not produce gaseous molecules through supersonic expansion, electrospray ionization yields molecular temperatures that are higher than the temperatures required for studying the spectroscopic properties in the molecular ground state. 

Therefore, it is beneficial to develop a new method for mildly evaporating molecules in the gas phase. In this study, we developed a new technology to mildly evaporate molecules from a solution using supersonic expansion. First, we attempted to evaporate the ionic species in water to convert them to the gas phase. We used an ultrasonic nebulizer because ultrasonic vibrators are used in ultrasonic humidifiers. Thus, we believed that an ultrasonic vibrator could convert the ionic species in water into the gas phase.

## 2. Results

**Figure 1:**
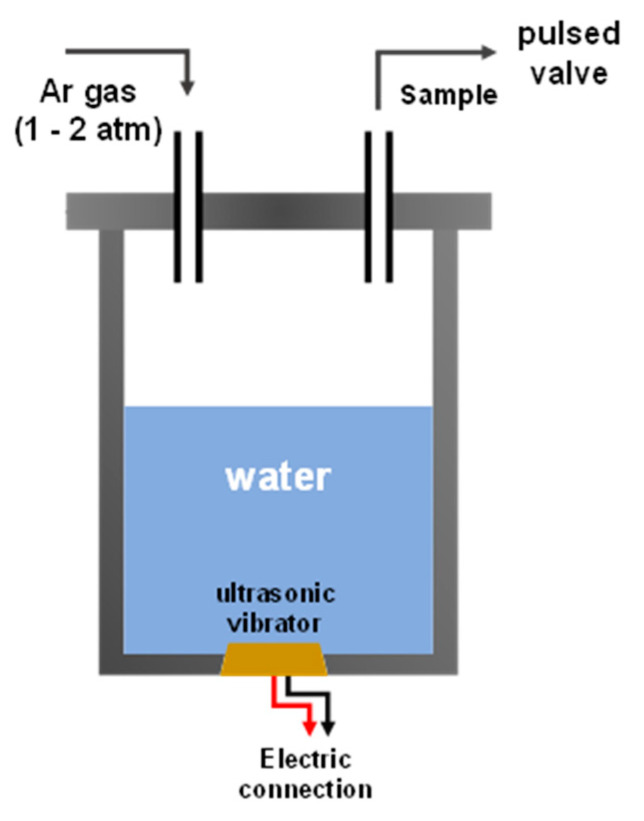
Design of sample container (aluminum) with ultrasonic vibrator (brown color). The top of container connects through gas tubes.

A humidifier produces water mist from liquid water; therefore, it is possible to evaporate hydrated molecules in the solution. Thus, we removed the ultrasonic vibrator from the ultrasonic humidifier and attached it to the bottom of a newly designed sample container, as shown in Figure 1. We then connected this sample container to an anion mass and photoelectron spectrometer (50 meV spectral resolution calibrated using O_2_ anion), to study the anionic molecules in the gas phase, the details of which have previously been reported [17,18]. 

**Figure 2:**
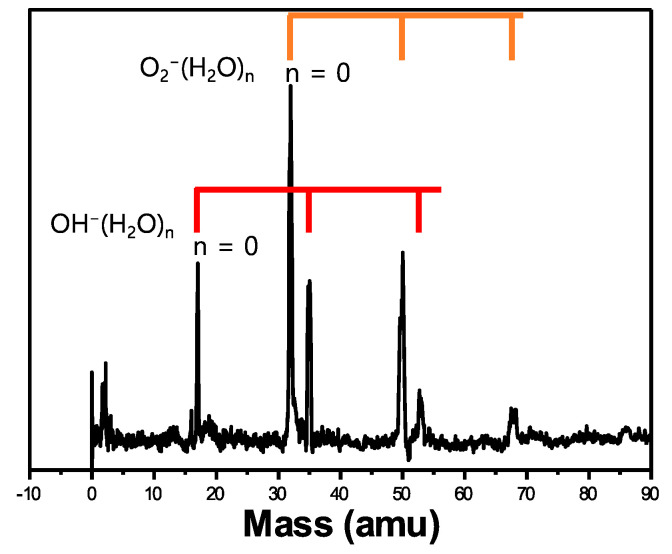
Mass spectra of OH^−^⋅(H_2_O)*_n_* and O_2_^−^⋅(H_2_O)*_n_*. Red line represents OH^−^**⋅** (H_2_O)*_n_* clusters and orange line indicates O_2_^−^⋅(H_2_O)*_n_*.

To evaluate the validity of this evaporation method, water was added to the sample container, and anionic-water-cluster formation was expected. Interestingly, stable hydroxide anions (OH^−^) were observed along with its water clusters, OH^−^⋅(H_2_O)*_n_* (*n* = 1–2), in the anion mass spectrometer, as shown in Figure 2. Alternatively, when increasing backing gas pressure (6 atm), mass peak intensities of each ion in a mass spectrum were differently observed as show in Figure S1. Lineberger and coworkers used microwave discharge to produce hydroxide anions in the gas phase [19,20]. The microwave discharge generated O^−^ anions, which reacted with NH_3_ molecules after the introduction of NH_3_ gas. Finally, OH^−^ and OH^−^⋅(NH_3_)*_n_* anion clusters were formed. Although these stable anionic clusters were observed in the mass spectrometer as well as in the photoelectron spectrometer, a complicated system was required to generate these anionic clusters. A two-step reaction generated hydroxide anions and their NH_3_ anionic clusters in the gas phase.

In our system, the origin of the OH^−^ anion is different from that in a previous study. We did not introduce any chemical reaction to generate hydroxide anions in the gas phase. Instead, our equipment directly generates hydroxide anions and their anionic clusters in water without any other chemical reactions. Two possible pathways could lead to the generation of anionic clusters. In the first pathway, OH^−^ anions are always in water, and thus, the ultrasonic nebulizer causes OH^−^ anions or OH^−^-anion–water clusters to directly evaporate from water. Using our equipment, we observed OH^−^ anions and their water clusters in the gas phase. The second pathway occurs when the ultrasonic nebulizer creates water molecules with a high internal temperature, which then collide with the electron beam and backing gas (Ar). This results in the dissociative reaction of water molecules, H_2_O → H^+^ + OH^−^. In addition, OH^−^⋅(H_2_O)_n_ clusters can be created from H_2_O·(H_2_O)*_n_* → H^+^ + OH^−^⋅(H_2_O)*_n_*. Although it is difficult to directly determine which pathway actually produces OH^−^ anions in the gas phase, we were able to stably observe these chemical species in the gas phase, and the molecular temperature was low enough (below 100 K) to observe their photoelectron spectrum. This indicates that there are no significantly intense vibrational hot bands in the photoelectron spectrum.

**Figure 3:**
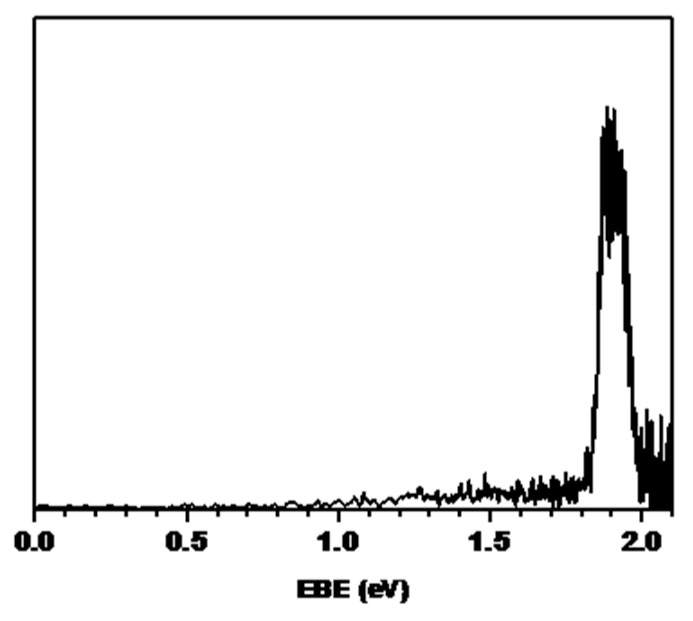
Photoelectron spectrum of OH^−^. The maximum peak in the spectrum is 1.90 ± 0.05 eV.

As shown in Figure 3, we obtained the photoelectron spectrum of the OH^−^ anion using a 532 nm laser. The anionic and neutral states displayed a good Franck–Condon overlap because the anionic and neutral structures were geometrically not very different: 0.965 Å for OH^−^ anion and 0.969 Å for OH neutral from the ab initio calculation at the MP2/6 −311 + + G** level using the Gaussian 16 package [21]. This resulted in the sharp spectral shape shown in Figure 3. The peak of the spectrum occurred at 1.90 ± 0.05 eV. Thus, the electron-binding energy of the OH^−^ anion was 1.90 ± 0.05 eV. This result is consistent with the findings reported by Lineberger et al. This indicates that our method is reliable for studying molecules in the gas phase and is less complicated than the two-step chemical-reaction method.

**Figure 4:**
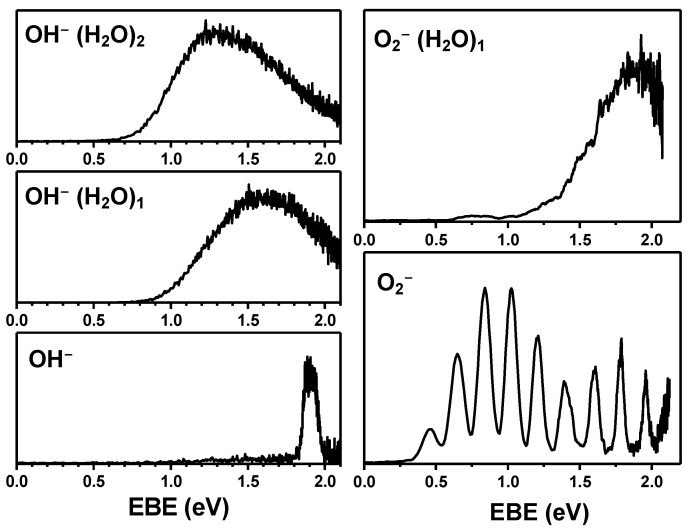
Photoelectron spectra of OH^−^ ⋅(H_2_O)*_n_* (*n* = 0–2) and O_2_^−^⋅(H_2_O)*_n_* (*n* = 0–1).

We also obtained the photoelectron spectra of OH^−^⋅(H_2_O)*_n_* (*n* = 1–2) clusters using a 532 nm laser. As shown in Figure 4, the vertical detachment energy (VDE) of OH^−^⋅(H_2_O)_1_ (1.50 ± 0.05 eV) was slightly lower than that of the OH^−^ anion (1.90 ± 0.05 eV). In addition, the spectral shape was much broader than that of OH^−^. In terms of spectral broadness, a higher molecular degree of freedom would have resulted in this broadness compared to the hydroxide anion. There would be more vibrational modes and therefore more unresolved vibrational modes contributing to spectral broadening. Similarly, the VDE of OH^−^⋅(H_2_O)_2_ was 1.30 ± 0.05 eV, which is lower than that of OH^−^⋅(H_2_O)_1_. The spectral shape is broader than that of OH^−^⋅(H_2_O)_1_. This broadness is also caused by many additional vibrational modes. To further reinforce our results, we acquired the photoelectron spectra of O_2_^−^ and O_2_^−^⋅(H_2_O)_1_, which also appeared in the mass spectrum. In terms of the spectral shape and electron-binding energies of these species, the spectra shown in Figure 4 are exactly the same as those in a previous report [22].

**Figure 5:**
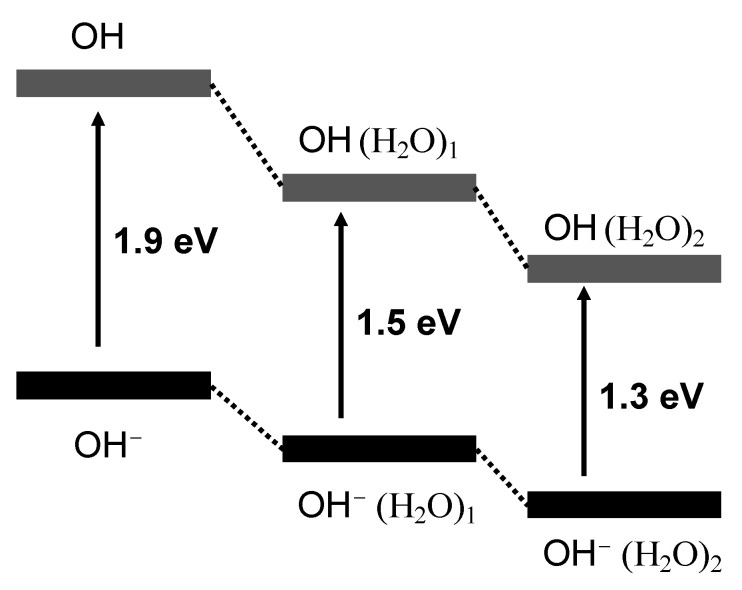
Energy diagrams of OH^−^ (H_2_O)*_n_* (*n* = 0–2).

Intriguing results concerned the electron-binding energy of the clusters. Normally, the electron-binding energy increases as a function of cluster size. When a molecule interacts with solvent molecules, the anionic state is typically much more stabilizing than the neutral state. As shown in Figure 4, the electron-binding energy of O_2_^−^⋅(H_2_O)_1_ shifted to higher binding energy than that of O_2_^−^. This is the basic concept of molecular solvation. Nevertheless, for the OH^−^⋅(H_2_O)*_n_* (*n* = 0–2) clusters, the electron-binding energies decreased when water molecules were added (Figure 5). 

## 3. Discussion

In general, anions are open-shell systems and neutrals are closed-shell systems. Thus, the solvation effect would increase electron-binding energy as a function of cluster size. The OH neutral radical is an open-shell system, whereas the OH^−^ anion is a closed-shell system. We observed the opposite of what we normally see. This led us to believe that the OH neutral radical was more polarizable than the OH^−^ anion. A comparison showed that the dipole moment of the neutral state was slightly higher than that of the anion. The photoelectron-spectrometer measurements validated these observations. 

In previous studies, the OH^−^ anion clustered with other molecules, such as NO_3_ and NH_3_, showing that electron-binding energies increased as a function of the size of the clusters. This agrees with what is normally observed for most anionic clusters. Our results were different than those reported in previous studies. Although our results are conceptually convincing, it is not clear why we observed the opposite behavior. To explain why the electron-binding energies decreased when the hydroxide anion contained more water molecules, we carried out various theoretical calculations (DFT -B3LYP, BP86 and BPBE- and HF, MP2 using various basis sets) using the Gaussian 16 package [21]. However, these calculations failed to explain our experimental findings. Contemporary methods have certain limitations in simulating accurate energetics. Thus, we believe that the development of theoretical models is required to enable the accurate calculation of the energetics of OH^−^ and its water clusters. Therefore, we performed further experiments with different chemical species to confirm the validity of our experimental results, as shown in Figure 4. Based on these results, we believe that our work provides valuable insights.

## 4. Method

The sample container was installed with an ultrasonic vibrator in the gas line between the pulsed valve and gas bottle in the photoelectron spectrometer. Thus, the evaporated molecules were cooled down in vacuum through supersonic expansion (backing gas pressure was 2–6 atm) using a pulsed valve. This allowed us to observe the molecular ground states of the evaporated molecules. The ultrasonic vibrator of an ultrasonic humidifier vibrates at a frequency of ≥20 kHz, and thus the vibrator pulsates and evaporates molecules in the solution. The molecular beam was created by supersonic expansion of the evaporated sample at approximately 2 atm pressure of Ar gas at room temperature.

## 5. Conclusions

We developed a new solution-evaporation system using an ultrasonic nebulizer. An ultrasonic vibrator was added to the bottom of the solution sample reservoir to enable the evaporation of the molecules to the gas phase. When we added this new sample-evaporation system to the supersonic-expansion equipment, the solution sample was easily converted to the gas phase, enabling observation of the OH^−^ anion and its water clusters. Although the photoelectron spectra of OH^−^⋅(H_2_O)*_n_* (*n* = 1–2) were mysterious, we could observe them in the mass spectrum as well as the photoelectron spectrum, giving rise to the possibility that this evaporation system can help convert biomolecules in solution to low-temperature molecules in the gas phase.

## Data Availability

The data taken in the study are all presented in the article.

## Supplementary Materials

The following are available online at https://www.mdpi.com/article/10.3390/ijms23084175/s1.
