# Supplementary material for: Photoelectron Spectroscopy of OH−-Anion–Water Clusters Generated by Ultrasonic Nebulizer
Source: Int J Mol Sci. 2022 Apr 10;23(8):4175. doi: 10.3390/ijms23084175 (PMC9030742; doi:10.3390/ijms23084175)
Supplement: Supplementary file 1 [file ijms-23-04175-s001.zip › ijms-1652968-supplementary.pdf]

Supplementary Materials of

Photoelectron spectroscopy of OH^--anion-water clusters generated by ultrasonic nebulizer

Minchae Kang [1], Chang Jun Park [1], Hyung Min Kim [2] and Sang Hak Lee [1,*]

[1] Department of Chemistry, Pusan National University, Busan 46241, Korea
[2] Department of Applied Chemistry, Kookmin University, Seoul 02707, Korea
[*] Correspondence: shlee@pusan.ac.kr; Tel.:+82-51-510-2245

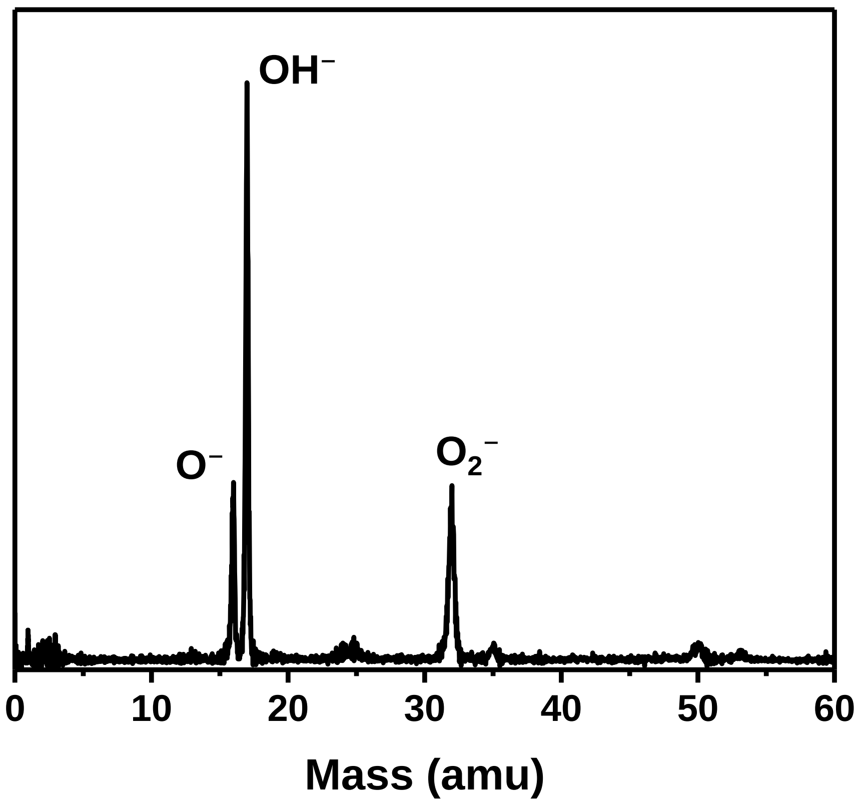

Figure S1. Mass spectrum of OH anion at high backing gas pressure (6 atm)

The mass spectrum was obtained at the higher backing gas pressure (6 atm). As compared with Figure 2 in the main article, we could not observe OH^- $(H_2O)_n$ clusters. We believe that this is attributed to high collision energy as well as the collision frequency with high-pressure Ar gas.
